# Trauma-informed neighborhoods: Making the built environment trauma-informed

**DOI:** 10.1016/j.pmedr.2021.101501

**Published:** 2021-07-20

**Authors:** Krista Schroeder, Jennie G. Noll, Kevin A. Henry, Shakira F. Suglia, David B. Sarwer

**Affiliations:** a Temple University College of Public Health – Department of Nursing, 3307 North Broad Street, Philadelphia, PA 11940, USA; b Penn State College of Health and Human Development – Department of Human Development and Family Studies, 325 Health and Human Development Building, University Park, PA 16802, USA; c Temple University College of Liberal Arts – Department of Geography and Urban Studies, 1115 Polett Walk, Philadelphia, PA 19122, USA; d Emory University Rollins School of Public Health – Department of Epidemiology, 1518 Clifton Road, Atlanta, GA 30322, USA; e Temple University College of Public Health – Department of Social and Behavioral Sciences, Center for Obesity Research and Education, 3307 North Broad Street, Philadelphia, PA 11940, USA

**Keywords:** Trauma, Trauma-informed care, Built environment, Neighborhood

## Abstract

Trauma is a response to a deeply distressing or disturbing event, such as sexual assault, violent crime, or childhood abuse. Trauma has impacted the majority of Americans, with racial/ethnic minority and socioeconomically disadvantaged populations being disproportionately affected. Extensive evidence demonstrates trauma’s harmful effects on physical and psychosocial functioning and healthcare costs. Over the past decade, there has been greater recognition of the need to respond to trauma across various care delivery and program settings. Such recognition led to development of trauma-informed care, an approach that acknowledges trauma’s widespread impact and delivers care in a manner to promote healing and avoid re-traumatization. To date, trauma-informed approaches have been applied to clinical interventions, social programs, and community organizations. However trauma-informed approaches have not been widely applied to the built environment. Here, we propose the concept of a trauma-informed neighborhood. The idea of a trauma-informed neighborhood has not been elucidated in public health or medicine, yet merits attention because physical aspects of the neighborhood environment, such as lighting, traffic density, noise, and greenspace, may either trigger trauma or promote healing for individuals and communities. Research using geospatial, population health, and community-engaged approaches is needed and could build from the existing literature on how the built environment impacts mental health. Evidence would have direct implications for public policy and urban planning, particularly for neighborhoods where residents bear a disproportionate trauma burden. Until the built environment is routinely included in trauma-informed efforts, a key setting that influences trauma recovery, health, and well-being will remain overlooked.

## Highlights

• Many individuals and communities have a history of trauma.
• Neighborhood built environments can trigger re-traumatization or foster healing.
• Trauma-informed approaches merit attention in urban design and public planning.
• Research is needed to explore the idea of trauma-informed neighborhoods.
• Trauma-informed neighborhoods may promote individual and community well-being.

Trauma impacts 62–90% of the adult population in the United States (Kilpatrick et al., 2013, Merrick et al., 2018), with members of racial/ethnicity minority and socioeconomically disadvantaged groups bearing a disproportionate burden (Merritt and Benningfield, 2019). The negative impact of trauma on physical and mental health is well established (Hughes et al., 2017, Pacella et al., 2013). Most approaches to understanding, addressing, and healing from trauma focus on an individual, family, or organizational setting; they typically overlook the built environment. However many physical aspects of a neighborhood may trigger trauma symptoms and promote re-traumatization, whereas others may foster healing. There is a need to elucidate the idea of trauma-informed neighborhoods in order to inform public policy and urban planning that can help alleviate the burden of trauma and promote well-being. Thus, the purpose of this commentary is to provide an introduction to trauma and trauma-informed care for the unfamiliar reader, to present the new concept of trauma-informed neighborhoods, and to identify research priorities.

## 1 Understanding trauma

Trauma is a response to a deeply distressing or disturbing event(s) that overwhelms an individual’s ability to cope, causes feelings of helplessness, and diminishes one’s sense of self and ability to feel the full range of emotions and experiences (Substance Abuse and Mental Health Services Administration, 2014). Traumatic experiences are not homogeneous and encompass a range of intensity, chronicity, and severity. However, general consensus exists on experiences that are often considered traumatic. For example, conception of trauma typically include childhood adversity, a broad term encompassing circumstances that threaten a child's physical or psychological well-being. Childhood adversity is often operationalized as “adverse childhood experiences” (ACEs), which include experiences such as physical, psychological, or sexual abuse, a family member’s substance use disorder, or witnessing intimate partner violence against a female caregiver (Felitti et al., 1998). Effects of childhood trauma often persist throughout the life course, given presence of sensitive developmental periods. Although childhood is a particularly vulnerable time, trauma also can occur during adulthood as well. While trauma was historically considered within the individual or household realm, recent evidence highlights the importance of neighborhood- and society-level factors, such as feeling safe in one’s neighborhood, experiencing discrimination, witnessing violent crime in the community, and historical trauma (trauma experienced by a cultural group resulting from marginalization, subjugation, loss, or oppression) (Conching and Thayer, 2019, Cronholm et al., 2015).

Response to trauma can vary greatly. Different kinds and levels of trauma can have different impact and the effect of multiple traumatic experiences is often synergistic rather than additive. For example, recent work illuminates how certain combinations of ACEs (particularly sexual abuse combined with other ACEs) interact to have disproportionately negative effects (Briggs et al., 2021). However, considered collectively, research is consistent in demonstrating trauma’s physiological, neuroendocrine, behavioral, and psychosocial harms. Populations who experience trauma - particularly multiple traumatic events or severe or chronic trauma - have a greater risk for cardiovascular disease, cancer, type II diabetes, and obesity (Felitti et al., 1998, Hughes et al., 2017, Noll et al., 2007, Norman et al., 2012, Pacella et al., 2013, Schroeder et al., 2021, Suglia et al., 2018). Neuroendocrine effects of trauma lead to heightened reactivity, hypervigilance, and hypothalamic pituitary axis dysregulation (Dunlop and Wong, 2019). Trauma negatively impacts mental health and psychosocial well-being, with increased risk for substance use disorder, depression, anxiety, suicide, and interpersonal violence (Hughes et al., 2017, Norman et al., 2012). Further, a subset of individuals who experience trauma develop post-traumatic stress disorder (PTSD), a mental health condition arising from traumatic events and resulting in persistent cognitive, behavioral, and mood-related symptoms (American Psychological Association, 2013).

## 2 Trauma-informed care

Trauma-informed care is an approach to incorporating key trauma principles into an organizational culture, in order to acknowledge the widespread impact of trauma, promote healing, and avoid re-traumatization (Substance Abuse and Mental Health Services Administration, 2014). Trauma-informed care is grounded in six principles: safety; trustworthiness and transparency; peer support; collaboration and mutuality; empowerment, voice, and choice; and cultural, historical, and gender issues (Substance Abuse and Mental Health Services Administration, 2014). Trauma-informed approaches have been applied widely in behavioral health, substance use, child welfare, social services, and community relationship building, with growing attention in certain healthcare settings such as emergency departments. In addition, trauma-informed design has been applied in select settings, such as correctional facilities (Covington and Bloom, 2007, Jewkes et al., 2019). However, trauma-informed approaches have not yet been applied to the neighborhood built environment.

## 3 Trauma-informed neighborhoods

A trauma-informed neighborhood is a neighborhood in which trauma-informed principles are adapted for and applied to the built environment. As of June 2021, there exists no manuscripts using the term “trauma-informed neighborhood” in PubMed or Google Scholar, suggesting that the idea has not yet been developed within academic circles. Trauma-informed approaches to the built environment are critically needed, given the clear impact of neighborhoods on health and the potential for physical neighborhood characteristics to either re-traumatize or promote healing, especially in neighborhoods where many residents have experienced trauma. Of note, the concept of trauma-informed neighborhoods is focused on translating established tenants of trauma-informed care to the physical built environment - an oft overlooked component of trauma efforts. The built environment is defined as “person-made or modified structures that provide people with living, working, and recreational spaces,” such as roads, greenspace, or building design (Environmental Protection Agency, 2020). Physical settings influence perceptions, behaviors, and experiences of individuals and communities. Social interactions, such as with family and peers, are influenced by physical environments and physical environments influence how social interactions are experienced (Chandrabose et al., 2019, Diez Roux, 2001, Foster et al., 2017, Johnson et al., 2018, Kärmeniemi et al., 2018, Malambo et al., 2016, McLeroy et al., 1988, Renalds et al., 2010, Sullivan and Chang, 2011). We posit that a trauma-informed built environment may promote well-being at the individual-level (e.g., increased feelings of safety), improve the social environment (e.g., greater community connectivity), and complement traditional person-centered efforts to address trauma. Such an approach would align well with the therapeutic landscape theories arising from medical geography, which center on the potential for physical spaces to be health promoting and healing environments (Bell et al., 2018, Gesler, 1992).

Evidence about how the built environment influences mental health would be relevant to the concept of trauma-informed neighborhoods. Numerous neighborhood conditions influence mental health (Truong and Ma, 2006). For example, limited personal outdoor access, less greenspace, and poor walkability are associated with depression. (Gong et al., 2016) Higher industrial use, increased traffic volume, and hazardous waste facilities are associated with stress and prescription of anxiolytics (Gong et al., 2016, Klompmaker et al., 2019, Matthews and Yang, 2010). Vacant, blighted urban land is associated with increased stress and anxiety (Garvin et al., 2013, Sivak et al., 2021). Neighborhood noise, artificial light exposure, and proximity to polluting traffic are associated with stress among youth; greenspace may buffer such effects (Dreger et al., 2015, Franklin et al., 2020). In addition, greenspace is associated with less psychological distress, less anger, and fewer prescriptions for psychiatric medications (Bowler et al., 2010, Klompmaker et al., 2019).

The tenants of trauma-informed care, combined with existing evidence on the relationship between neighborhoods and mental health, suggest pathways by which a trauma-informed built environment may influence individual well-being and the social environment. For example, many urban neighborhoods have sidewalks bordering dangerous traffic patterns, poorly lit dark alleys, and loud noises (e.g., jackhammering from construction in gentrifying neighborhoods, loud horns from busy roads, police sirens). All could cause distress, particularly for those with a history of trauma. In contrast, noise barriers or construction start time restrictions could promote feelings of safety. Many neighborhoods lack pleasant outdoor spaces. Greenspace may be comprised of poorly maintained, litter-filled grass lots or well-kept gardens with lush greenery. Certain building characteristics may foster feelings of threat (e.g., metal bars over windows, razor wire, police cameras); in contrast, community-provided murals may contribute to cohesion. Neighborhood residents are often excluded from decision-making about use and modification of their built environment, yet involving residents could promote their empowerment and collaboration.

### 3.1 Next steps and future research

There is an opportunity for research that elucidates the concept of trauma-informed neighborhoods. Studies are needed to understand how a trauma-informed neighborhood can promote individual well-being, benefit the social environment, and complement person-centered trauma interventions. Trauma-informed neighborhood research should take a community-engaged approach and not assume that researchers know what aspects of the built environment are potentially traumatizing. A population health approach would be needed to translate how principles at higher levels of the ecological model (built environment) can be applied to a concept often considered at the individual level (trauma). Geospatial studies could take advantage of growing neighborhood-level data sources to disentangle which neighborhood factors are associated with trauma (Rundle et al., 2011). The potential to build consideration of trauma-informed neighborhoods into individual-level therapeutic interventions is another area for future research, as is exploration of related concepts, such as environmental trauma experienced by children who have been exposed to lead via failures in civic planning and urban policy.

Of note, neighborhoods where many residents have experienced trauma may benefit most from a trauma-informed approach. Targeted approaches can help illuminate which ACEs are most salient to specific neighborhoods. Yet, when focusing research in neighborhoods with greatest need, sensitivity would be necessary to ensure research does not perpetuate and actively works to dispel stigma associated with trauma. (Kwan, 2012) Importantly, while there is overlap with research on how the built environment impacts mental health, trauma-informed neighborhood research would need to maintain focus on *trauma*, including how tenants of trauma-informed care relate to the built environment and how the built environment can worsen trauma-related symptoms or instead promote healing.

## 4 Conclusion

By ignoring the built environment, efforts to address trauma lack a comprehensive, ecological approach. There exists a need to translate trauma-informed efforts to the physical neighborhood environment. Work elucidating a trauma-informed neighborhood would have direct implications for public policy and urban planning related to the built environment. Efforts to build the evidence for trauma-informed neighborhoods could support health and well-being of those who have experienced trauma in neighborhoods around the world.

## Funding

This research was funded by the 10.13039/100009633Eunice Kennedy Shriver National Institute of Child Health and Human Development (grant number: K23 HD101554; PI: Schroeder) of the National Institutes of Health (NIH). Dr. Sarwer’s work was supported by grant funding from the National Institutes of Health (National Institute for Diabetes, Digestive, and Kidney Disease R01 DK108628 and 10.13039/100000072National Institute of Dental and Craniofacial Research R01 DE026603) as well as PA CURE Funds from the Commonwealth of Pennsylvania. The content is solely the responsibility of the authors and does not necessarily represent the views of the NIH. The funders had no role in the development or preparation of this manuscript.

## Declaration of Competing Interest

The authors declare that they have no known competing financial interests or personal relationships that could have appeared to influence the work reported in this paper.
